# Map-based cloning and characterization of *BoCCD4*, a gene responsible for white/yellow petal color in *B. oleracea*

**DOI:** 10.1186/s12864-019-5596-2

**Published:** 2019-03-25

**Authors:** Fengqing Han, Huilin Cui, Bin Zhang, Xiaoping Liu, Limei Yang, Mu Zhuang, Honghao Lv, Zhansheng Li, Yong Wang, Zhiyuan Fang, Jianghua Song, Yangyong Zhang

**Affiliations:** 1 Institute of Vegetables and Flowers, Chinese Academy of Agricultural Sciences, Key Laboratory of Biology and Genetic Improvement of Horticultural Crops, Ministry of Agriculture, #12 Zhong Guan Cun Nandajie Street, Beijing, 100081 China; 2 College of Horticulture, Anhui Agricultural University, Hefei, 230036 China

**Keywords:** *Brassica oleracea*, Petal color, Fine mapping, Candidate gene, Functional validation, Expression pattern

## Abstract

**Background:**

*Brassica oleracea* exhibits extensive phenotypic diversity. As an important trait, petal color varies among different *B. oleracea* cultivars, enabling the study of the genetic basis of this trait. In a previous study, the gene responsible for petal color in *B. oleracea* was mapped to a 503-kb region on chromosome 3, but the candidate gene has not yet been identified.

**Results:**

In the present study, we report that the candidate gene was further delineated to a 207-kb fragment. *BoCCD4*, a homolog of the *Arabidopsis carotenoid cleavage dioxygenase 4* (*CCD4*) gene, was selected for evaluation as the candidate gene. Sequence analysis of the YL-1 inbred line revealed three insertions/deletions and 34 single-nucleotide polymorphisms in the coding region of *BoCCD4.* Functional complementation showed that *BoCCD4* from the white-petal inbred line 11–192 can rescue the yellow-petal trait of YL-1. Expression analysis revealed that *BoCCD4* is exclusively expressed in petal tissue of white-petal plants, and phylogenetic analysis indicated that CCD4 homologs may share evolutionarily conserved roles in carotenoid metabolism. These findings demonstrate that *BoCCD4* is responsible for white/yellow petal color variation in *B. oleracea*.

**Conclusions:**

This study demonstrated that function loss of *BoCCD4*, a homolog of *Arabidopsis CCD4*, is responsible for yellow petal color in *B. oleracea*.

**Electronic supplementary material:**

The online version of this article (10.1186/s12864-019-5596-2) contains supplementary material, which is available to authorized users.

## Background

The flower is the reproductive structure of angiosperms, and petals exhibit extensive color variation, mainly due to the accumulation of flavonoids, carotenoids and/or betalain pigments. Flower color serves as a visual signal to attract pollinators and is thus very important for plant reproduction [1–3]. There are several reports that by affecting gene exchange, changes in flower color contribute to the species differentiation [4, 5]. Thus, flower can serve as a model for studying the relationship between phenotype and genotype during evolution [6]. Additionally, flower color protects plants against disease and UV radiation and helps to maintain the normal physiological function of floral organs [7, 8].

Carotenoids are mostly C_40_ isoprenoid compounds, comprising of over 750 members widely distributed in fungi, cyanobacteria, algae and plant [9]. Carotenoids biosynthesis takes place in plastids of plants. They present in photosynthetic tissues for light harvesting and photoprotection during photosynthesis [10]. In non-photosynthetic tissues, carotenoids impart color ranging from yellow to red to fruits and flowers as well as other organs [11]. Carotenoids also provide precursors for biosynthesis of plant hormones, including abscisic acid (ABA) and strigolactones [12, 13]. The pathway of carotenoid biosynthesis has been well characterized, and nearly all the enzymes involved in carotenoid biosynthesis in plants have been identified (see reviews by Howitt and Pogson; Ruiz-Sola and Rodríguez-Concepción) [14, 15].

Carotenoids are catabolized to produce various apocarotenoids by an evolutionarily conserved carotenoid cleavage dioxygenases (CCDs). In the model plant *Arabidopsis*, the CCDs family has 9 members that are divided into two groups: five 9-cis-epoxycarotenoid dioxygenases (NCED2, NCED3, NCED5, NCED6 and NCED9) and four CCDs (CCD1, CCD4, CCD7 and CCD8) [16, 17]. NCEDs specifically cleave 9-cis-violaxanthin and 9-cis-neoxanthin to produce C_15_ xanthoxin, the precursor for ABA [18]. CCDs act on different substrates, and their precise roles are not fully understood. CCD1 and CCD4 have multiple substrates, catalyzing carotenoid cleavage at different double-bond positions to produce such compounds as α-ionone, β-ionone, β-cyclocitral, crocin [17, 19], some of which attribute to scent and flavor of plant flowers and fruit [20, 21]. Moreover, CCD1 and CCD4 may be responsible for fruit and flower color variation in some species by affecting the accumulation of colorful carotenoids [17, 22–24]. Recent studies have found that CCD4 homologs play key roles in determining the white or yellow color of flowers in *Chrysanthemum* [25], *Brassica napus* [17] and azalea [23], and fruit color in peach [24]. CCD7 and CCD8 act sequentially in the strigolactone pathway by cleaving β-carotene to produce the precursor of strigolactone [26], a hormone regulating plant shoot branching and nodulation [27].

*Brassica oleracea* comprises multiple subspecies showing extreme phenotypic diversity. As an important trait, flower color varies among different *B. oleracea* cultivars: pervasive yellow petals with different degrees of yellowness and relatively fewer white petals, only existing in some cultivars of Chinese kale and cauliflower. Biochemical studies have revealed that variation in flower color in *Brassica* species is due to differences in the presence, amount, or type of carotenoid pigment [17, 22, 28]. Although previous studies have demonstrated that this yellow/white petal trait in *B. oleracea* is controlled by a single locus on C03 [29–31], the candidate gene has not yet been identified, and the molecular mechanism underlying petal color variation in *B. oleracea* species has not been elucidated.

Previously, we mapped the gene *cpc-1* responsible for petal color in *B. oleracea* to a 503-kb region [31], though the candidate gene was not found. In the present study, we reported further mapping results for *cpc-1*. The coding region of the candidate gene was cloned and compared between white-petal line 11–192 (a Chinese kale inbred line) and yellow-petal line YL-1 (a cabbage inbred line). *Agrobacterium*-mediated transformation of *B. oleracea* was conducted to validate the function of the candidate gene.

## Results

### Fine mapping of the petal color gene *cpc-1*

In a previous study, the candidate gene for petal color was mapped to a 503-kb region on C03 [31]. A larger F_2_ population was then developed, with 1251 recessive (yellow petal) individuals. By genotyping all 1251 recessive individuals using two flanking markers, we obtained 36 recombinants for M4064 and 22 recombinants for M4139. To genotype all recombinant individuals, additional InDel markers were developed in this interval. However, we detected a possible error in the 02–12 assembly (http://www.ocri-genomics.org/bolbase/index.html), as several markers showed different orders in the genetic map compared with their physical positions.

**Fig. 1:**
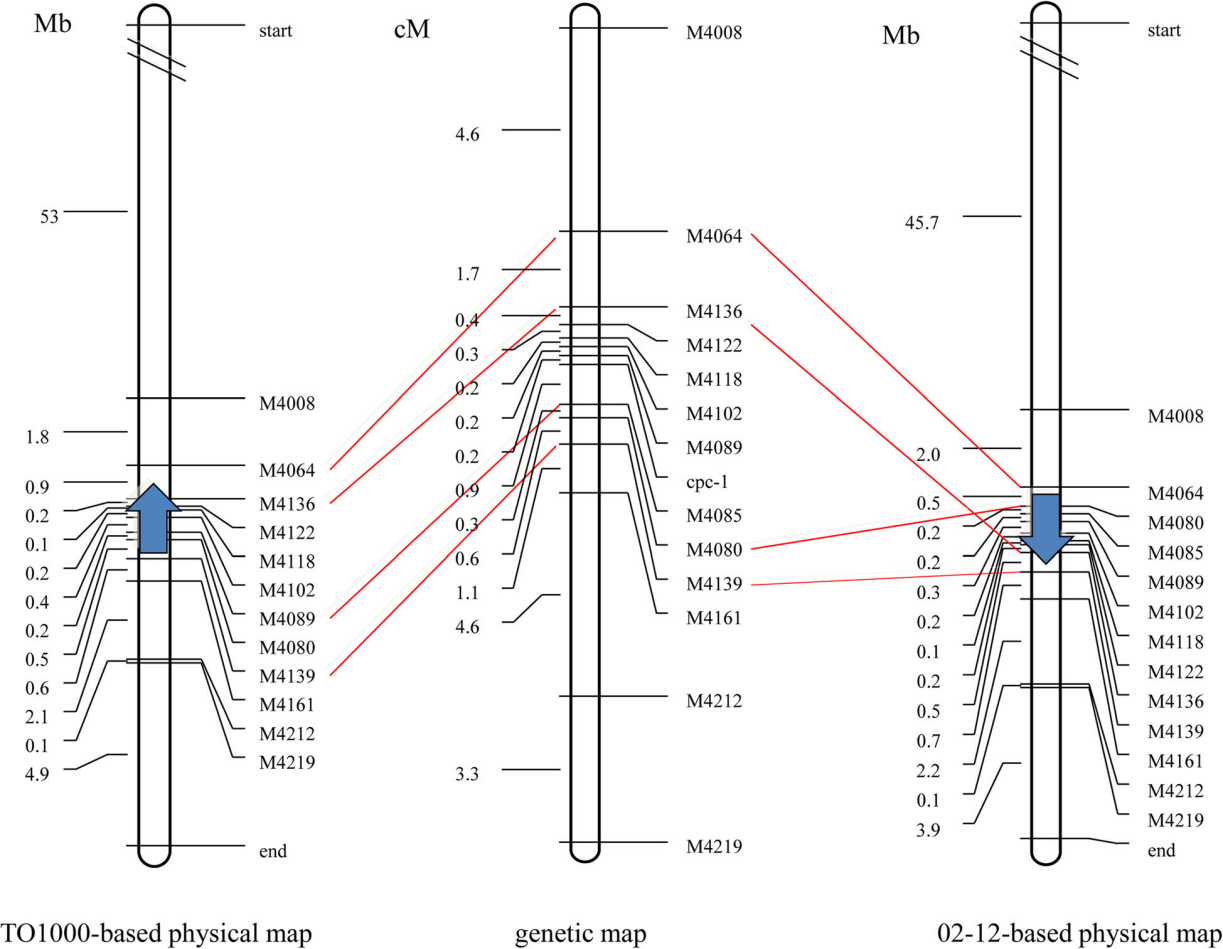
Map-based cloning of the *B. oleracea* gene *cpc-1*. The genetic map is in the middle; all markers are in the same order as in the TO1000-based physical map, but six markers are in reverse order compared with the 02–12-based physical map. Blue arrows indicate Scaffold000063

Thus, another physical map was constructed based on the TO1000 reference genome (http://plants.ensembl.org/Brassica_oleracea/Info/Index). By comparing the mapping region in the 02–12 and TO1000 reference genomes, we found that the region spans two scaffolds, Scaffold000063 and Scaffold000205, in the 02–12 reference genome. The physical and genetic maps indicated that Scaffold000063 was reversely assembled. In addition, *cpc-1* was re-mapped to a 207-kb genomic region (C03:48,444,077..48,651,173) flanked by markers M4089 and M4085, with genetic distances of 0.16 cM and 0.88 cM, respectively. The genetic and physical maps are shown in Fig. 1.

### *Bol029878* is the candidate gene for *cpc-1*

*B. oleracea* database (http://brassicadb.org/brad/) analysis revealed 14 predicted genes (Table 1) in the 207-kb region. *Bol029878* is a homolog of *Arabidopsis CCD4*. Due to its important role in oxidative cleavage pathways of carotenoids, *Bol029878* was chosen as a candidate gene and named *BoCCD4*.

**Table 1:** The 14 putative gene models in the target mapping region

Gene ID	Location	Homologous gene in *A. thaliana*	Annotation
*Bol029866*	C03: 48450632: 48454045	*AT4G19510*	disease resistance protein
*Bol029867*	C03: 48454444: 48458688	*AT4G19450*	general substrate transporter, nodulin-related
*Bol029868*	C03: 48463969: 48465140	*AT4G19440*	pentatricopeptide repeat-containing protein
*Bol029869*	C03: 48465153: 48465785	*AT4G19440*	pentatricopeptide (PPR) repeat-containing protein
*Bol029870*	C03: 48524222: 48527614	*AT4G19420*	pectinacetylesterase family protein
*Bol029871*	C03: 48555809: 48558510	*AT4G19410*	pectinacetylesterase
*Bol029872*	C03: 48580331: 48580891	*AT4G19360*	SCD6 protein-related
*Bol029873*	C03: 48582181: 48583206	*AT4G19360*	SCD6 protein-related
*Bol029874*	C03: 48587477: 48588334	*AT3G42170*	glycoside hydrolase
*Bol029875*	C03: 48592039: 48594751	*AT4G19210*	transporter; ATP-binding
*Bol029876*	C03: 48604615: 48605682	*AT4G19200*	proline-rich family protein
*Bol029877*	C03: 48612702: 48614889	*AT4G19185*	integral membrane family protein
*Bol029878*	C03: 48631489: 48640512	*AT4G19170*	carotenoid oxygenase; NCED4 (NINE-CIS-EPOXYCAROTENOID DIOXYGENASE 4)
*Bol029879*	C03: 48649269: 48651264	*AT4G19160*	unknown protein

**Fig. 2:**
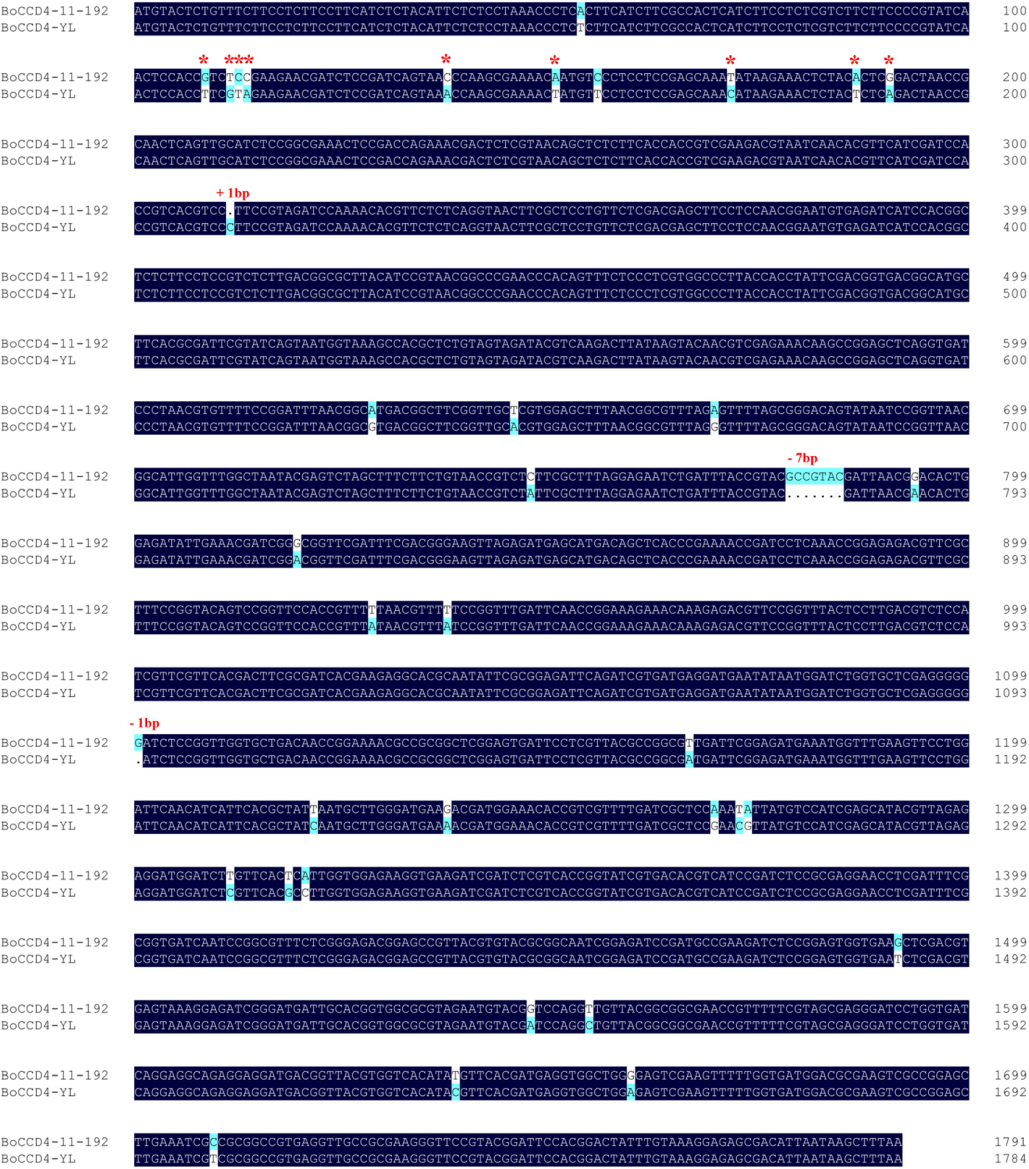
Sequence alignment of *BoCCD4* from 11-192 and YL-1. *BoCCD4* from YL-1 shows 3 InDels and 34 SNPs compared with 11–192. Red asterisks indicate the non-synonymous SNP mutations

The full-length sequence of the *BoCCD4* gene was downloaded from two reference genomes, TO1000 and 02–12. Wild-type *BoCCD4* has one exon predicted to encode a putative 596-amino acid protein, with 87.1% sequence identity with *Arabidopsis* CCD4. To detect any nucleotide variation in *BoCCD4* between white- and yellow-petal plants, the region encompassing the gene body and − 2-kb promoter of *BoCCD4* was amplified and sequenced using genomic DNA from YL-1 and 11–192. The gene sequence from 11-192(GenBank no. MK599257) was identical to that of TO1000, consistent with the fact that TO1000 is a Chinese kale-like-morphology plant with white flowers. Sequencing of this gene revealed multiple mutations in YL-1 (GenBank no. MK599258), a 1-bp insertion at the + 312 nucleotide position, a 7-bp deletion at the + 771 nucleotide position, a 1-bp deletion at the + 1094 nucleotide position, and 34 single-nucleotide polymorphisms (SNPs) (Fig. 2). All InDels result in a frameshift and premature stop codon. As the first important mutation in the coding region, the 1-bp insertion alters the open reading frame from the 105th position and causes a premature stop codon, resulting in a predicted truncated 114-amino acid protein lacking the important carotenoid-oxygenase domain (amino acids 74–590) (Additional file 1). Using first-strand cDNA as a template, amplification with the primer Bocpc-CDS generated a full-length coding sequence of *BoCCD4* from 11 to 192 but no product from YL-1. Together with RT-PCR results (see below), this finding indicates that *BoCCD4* is not expressed in yellow-petal line YL-1, possibly due to altered transcript stability [32] or mutations in the promoter region. Indeed, we detected mutations in the − 2-kb promoter region, though it remains to be determined whether these mutations are crucial for suppressing expression of the transcript.

A genic marker, Bol035718D771, was designed based on the 7-bp deletion and used for genotyping the parental line and recombinants of M4089 and M4085. All 13 recombinants showed the same band size as that of YL-1 (Additional file 2), indicating that this gene was co-segregates with the petal color phenotype.

### Expression pattern of *BoCCD4*

**Fig. 3:**
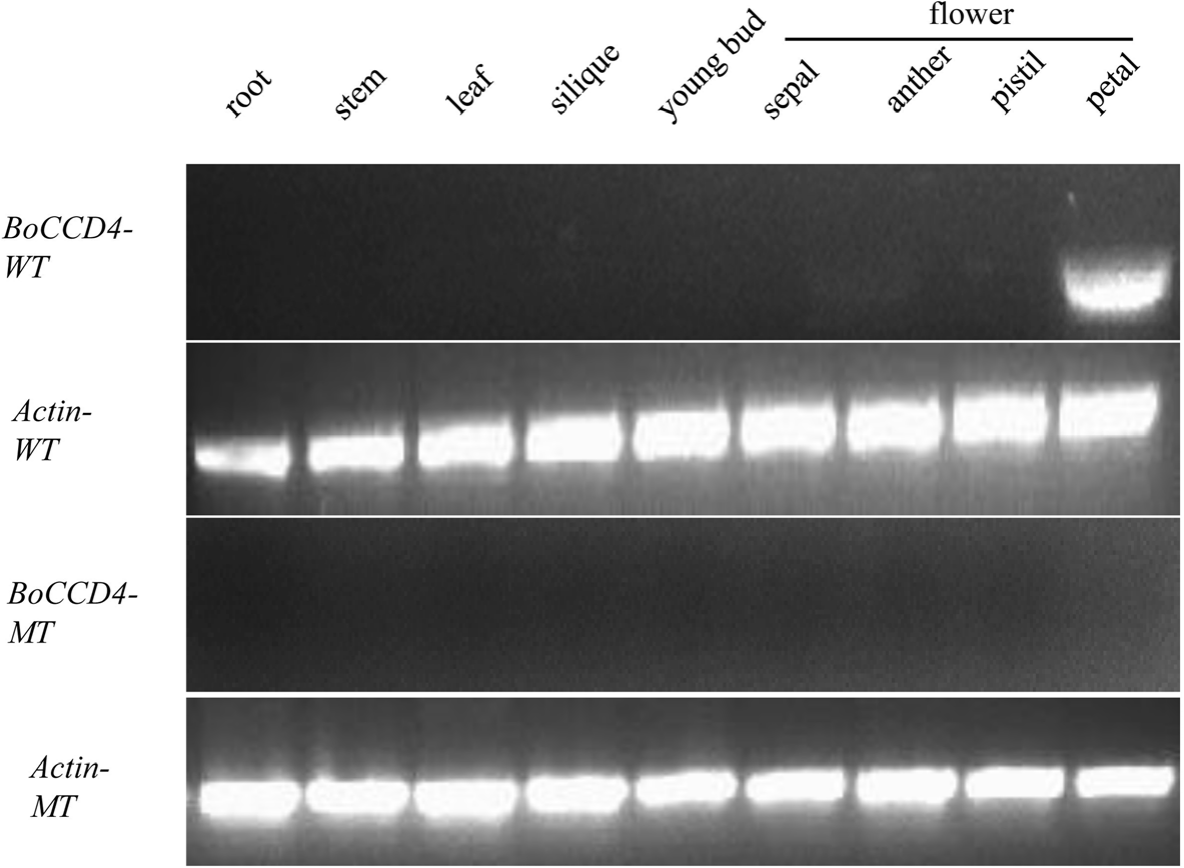
Expression pattern of *BoCCD4*. *BoCCD4* was exclusively expressed in petal tissue of white-petal plants. BoCCD4-WT, the wild type *BoCCD4* from 11-192 (white-petal inbred line); BoCCD4-MT, the mutant type *BoCCD4* from YL-1 (yellow-petal inbred line); Actin-WT, actin from 11-192; Actin-MT, actin from YL-1

To analyze the expression pattern of *BoCCD4*, semiquantitative RT-PCR was performed using different tissues: root, stem, leaf, silique, young buds and anther, pistil and open-flower petal. No expression was detected in any of the YL-1 tissues. However, *BoCCD4* was preferentially expressed in petals of the white-petal line (Fig. 3). These results revealed that *BoCCD4* is a tissue-specific gene that may cleave carotenoids in floral tissues, which is very different from its homolog in *Arabidopsis*.

### Overexpression of *BoCCD4* in YL-1 results in a transition of petal color from yellow to white or pale yellow

**Fig. 4:**
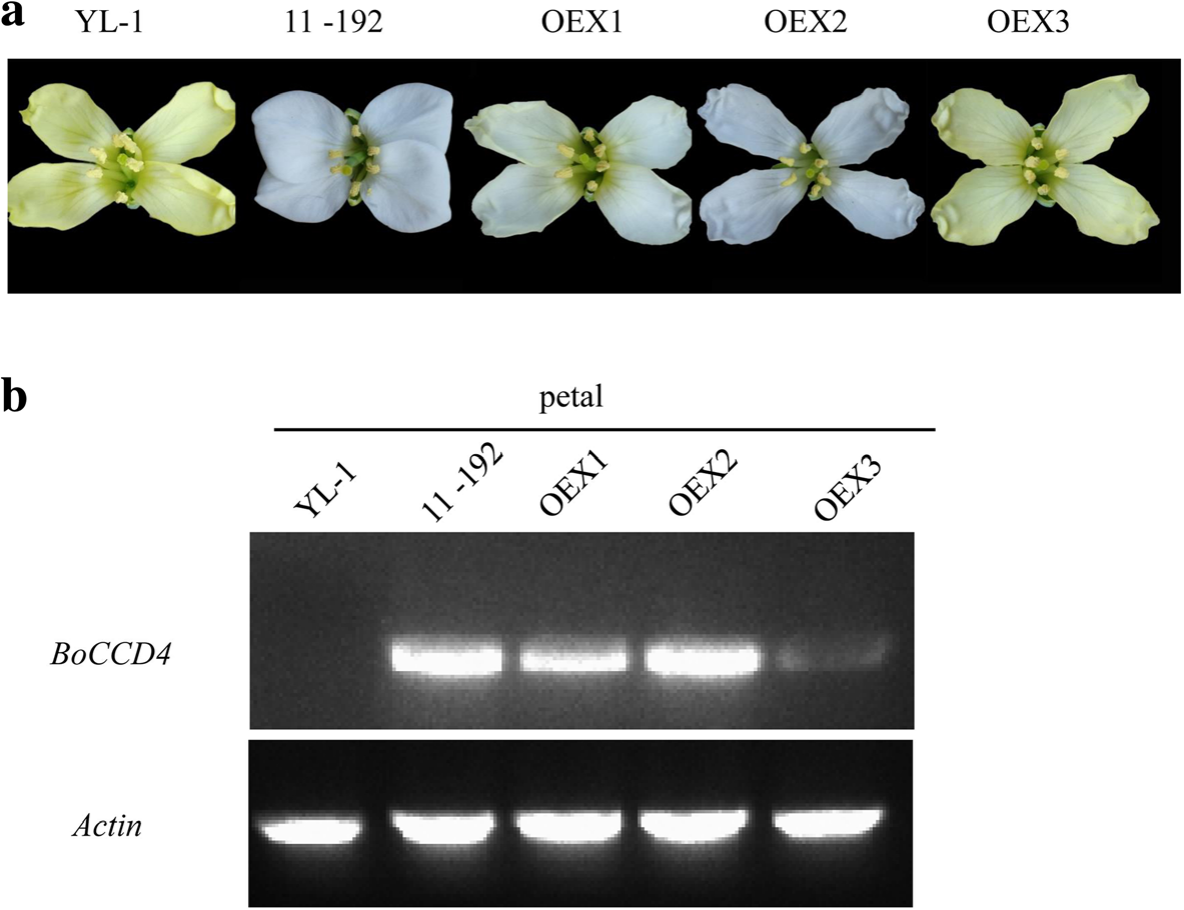
Phenotype of parental lines and three overexpressing transgenic lines. **a** The overexpressing transgenic lines OEX1 and OEX3 showed an intermediate phenotype; OEX2 displayed a completely white petal with no difference from that of 11–192. **b** expression level of *BoCCD4* in parental lines and three overexpressing transgenic lines. Among the overexpressing transgenic lines, OEX2 showed the highest expression level, followed by OEX1 and OEX3, with a high correlation with phenotype

We introduced wild-type *BoCCD4* driven by the CaMV35S promoter into the yellow-petal parent YL-1 using *Agrobacterium*-mediated *B. oleracea* transformation and obtained three independent overexpressing transgenic lines, OEX1, OEX2 and OEX3. OEX1 and OEX3 showed intermediate phenotypes, whereas OEX2 displayed a completely white petal similar to that of the white-petal line 11–192 (Fig. 4a).

We next examined the expression levels of the *BoCCD4* gene introduced into these transgenic lines by semiquantitative RT-PCR. OEX2 showed the highest expression level, followed by OEX1 and OEX3 (Fig. 4b), indicating high correlation between the expression level of *BoCCD4* and the white-petal phenotype. These results suggest that *BoCCD4* disruption is responsible for yellow petal color in *B. oleracea*.

### Phylogenetic analysis

**Fig. 5:**
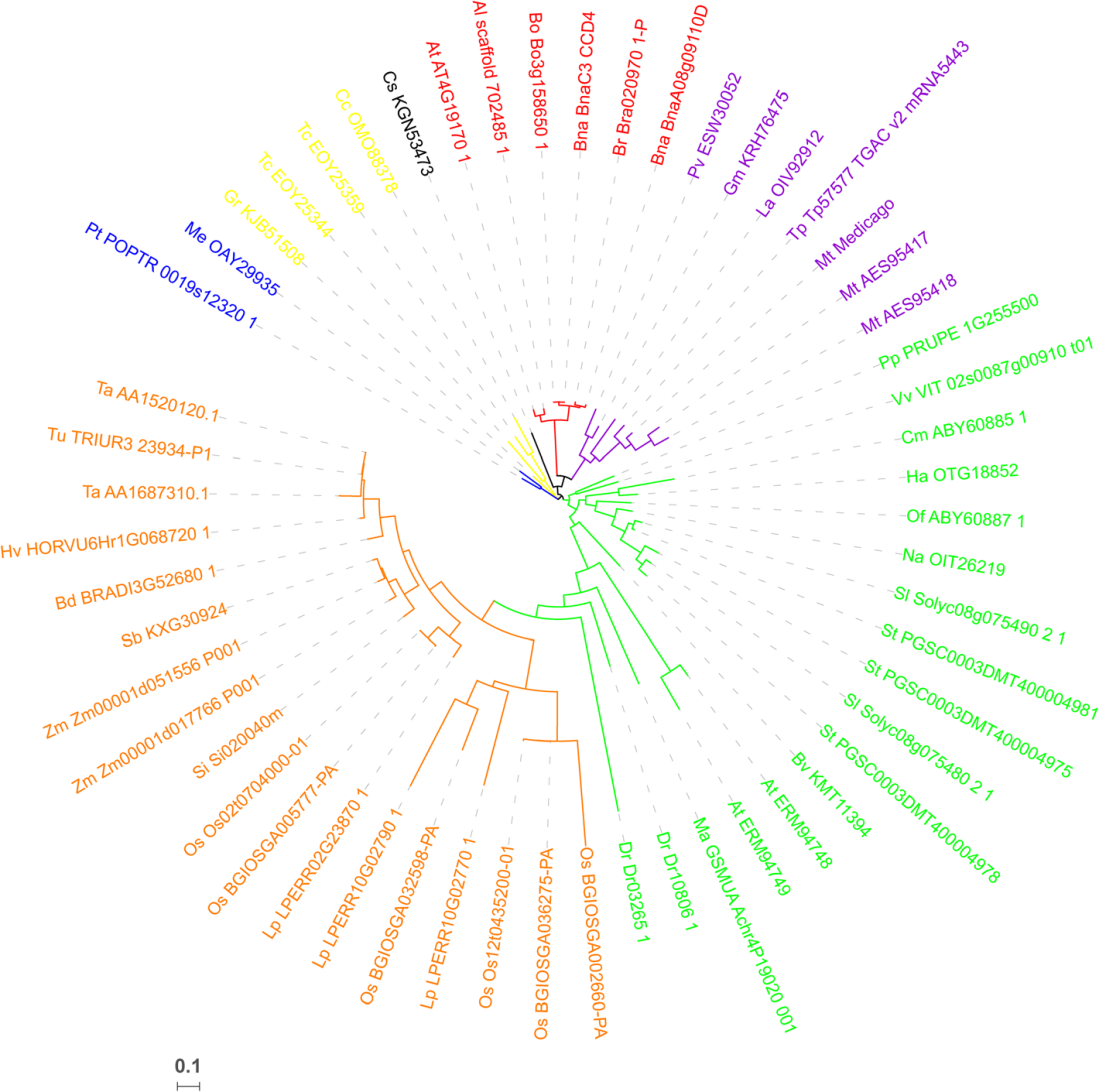
Phylogenetic analysis of *BoCCD4* and its related proteins. The analyses involved 55 *BoCCD4* homologs from 38 species

**Fig. 6:**
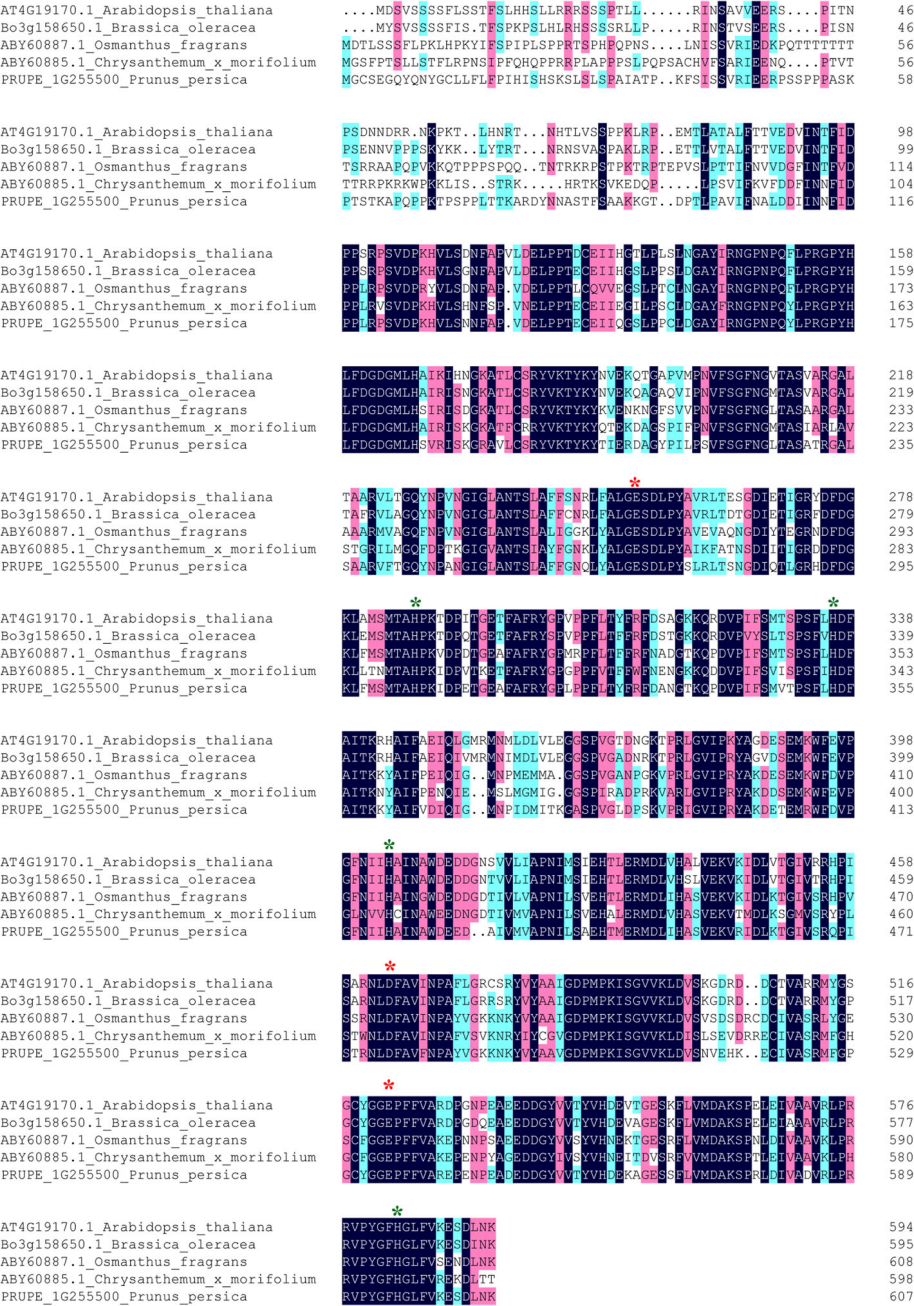
Sequence alignment of the BoCCD4 amino acid sequence and four functionally characterized CCD4s from *Arabidopsis thaliana*, *Osmanthus fragrans*, *Chrysanthemum x morifolium*, *Prunus persica.* Green asterisks indicate the four highly conserved histidine residues as an iron-ligating cofactor; red asterisks indicate the conserved glutamates or aspartate for fixing the iron-ligating histidine residues

To analyze the phylogenetic relationship between the BoCCD4 protein and its close homologs, we conducted BLASTP searches based on the protein database of NCBI and Ensembl Plants (http://plants.ensembl.org) using the full-length amino acid sequence of BoCCD4. We generated a neighbor-joining tree comprising BoCCD4 and 55 homologs from 38 species. These homologs were grouped into three main clades. BoCCD4 shows 87.1% sequence identity with *Arabidopsis* CCD4 and is located in the same clade as *Arabidopsis* CCD4, along with homologs from other cruciferous plants, *B. rapa* and *B. napus* (Fig. 5). All CCD4s from Cruciferae species evolved from a common ancestor. *Arabidopsis* has one CCD4, wheareas Brassica species retained two CCD4s homologs. Brassica CCD4s were assigned to subclades in accordance with their locations on A, B or C genome, indicating that Brassica CCD4s rapidly evolved after the whole-genome duplication event and the Brassiceae-lineage-specific whole-genome triplication event. We also conducted sequence alignment and analyses using BoCCD4 and functionally characterized CCD4s in other species, including *Arabidopsis thaliana*, *Osmanthus fragrans*, *Chrysanthemum x morifolium*, and *Prunus persica* (Fig. 6)*.* All of these CCD4s contain a chloroplast transient peptide, four highly conserved histidine residues as an iron-ligating cofactor, and a glutamates or aspartate residue for fixing the iron-ligating histidines. These results indicate a conserved role for CCD4s in carotenoid metabolism.

## Discussion

In *B. oleracea*, the white-petal trait segregates as a single locus, and white is dominant over yellow confirmed by different crosses [29–31, 33]. Recently, this locus was mapped to a region on C03 [29, 31]. In this study, we narrowed the gene to a 207-kb region, and we identified an incorrectly assembled scaffold, Scaffold000063, in the 02–12 genome, making positional mapping difficult. Two *B. oleracea* draft genome sequences are currently available: TO1000 (Chinese kale like) [34], and 02–12 (cabbage) [35]. These draft genomes facilitate basic genetics and genomics research but still need to be improved. The *B. oleracea* genome is estimated to be over 600 Mb, though the published pseudo-chromosome size is 388.8 Mb for 02–12 and 488.6 Mb for TO1000 [34, 35]. *B. oleracea* genome assembly errors are apparently not rare in previous studies [36–38]. In particular, regarding Lee et al., a genotyping-by-sequencing-based high-resolution genetic map allowed identification of 37 misanchored scaffolds for 02–12 and 2 misanchored scaffolds for TO1000 [38].

We predicted a carotenoid cleavage dioxygenase gene, *BoCCD4,* homologous to the *Arabidopsis CCD4* gene as the candidate gene. Sequence analysis, functional complementation, and expression pattern analysis demonstrated that functional loss of *BoCCD4* has resulted in widespread yellow-petal *B. oleracea* accessions. A similar *CCD4*-based mechanism has been found in other plants. In chrysanthemum (*Chrysanthemum morifolium Ramat*.), *CmCCD4a* degrades carotenoids into colorless compounds, resulting in a white petal color, as confirmed by expression and RNA interference (RNAi) analyses [25]. In azalea (*Rhododendron japonicum f. flavum*), high expression of a *CCD4* gene was identified in a white-flowered accession and its progeny and is considered the key factor controlling flower color [23]. In peach (*P. persica*), evidence from cultivars, somatic revertants and ancestral relatives support that *PpCCD4* is responsible for white/yellow flesh color and that yellow peach alleles have arisen from three independent mutations [32]. In *B. napus*, Zhang et al. reported that a transposable element insertion (TE1) disrupts *BnaC3.CCD4*, resulting in a yellow flower [17]. TE1 was also identified in some accessions of *B. oleracea*, for example, in cabbage lines 02–12 (draft genome) and some yellow-petal Chinese kale lines, indicating that flower petal color variation in *B. oleracea* follows a similar *CCD4*-disruption mechanism, as confirmed in the present study. Additionally, it is possible that yellow petals originally appeared in ancestors of *B. oleracea* and that one mutant type, i.e. TE1, was passed to *B. napus*.

*BoCCD4* is a floral tissue-specific gene that differs from *Arabidopsis CCD4* which is expressed in various vegetative tissues and floral tissues. *B. oleracea* has experienced a whole-genome duplication (WGD) event [39–41] and subsequent whole-genome triplication (WGT) [41]. According to previous studies, duplicated gene copies may undergo divergence in expression patterns or functions [41, 42]. It is interesting that one of the duplicated copies, *BoCCD4* on C03, evolved tissue-specific expression patterns and underwent loss-of-function events, converting flower color from white to yellow without influencing carotenoid metabolism in vegetative tissues. This phenomenon has also been found in other plants, whereby duplicated *CCD4* genes evolved different expression patterns in tomato [43], *C. morifolium* [25], and mandarin orange [44]. In addition, Rodrigo et al. reported that one *CCD4* copy evolved novel carotenoid cleavage activity [44].

Parallel evolution is a common evolutionary phenomenon in which different populations independently evolve the same trait [45, 46]. For example, three dwarf populations of the forest tree *Eucalyptus globulus* have evolved in parallel from local tall ecotypes [47]. In the Mina lineage of *Ipomoea*, parallelism was observed at different levels during the transition of flower color, primarily caused by cis-regulation of the *F3’H* gene [48]. In addition, parallel evolution at the *FLC* locus has conferred flowering time variation in the cruciferous plant *Capsella rubella*. In *B. oleracea*, using different accessions, we confirmed the presence of at least five pervasive key mutations in the coding region of *BoCCD4*: two transposons (TE1 and TE2) and three InDels (+ 312 insertion, + 771 deletion, and + 1094 deletion). Different yellow-petal haplotypes (nonfunctional alleles) harbor one independent key mutation or combination of two or more mutations. The presence of these independent mutations indicates that parallel evolution of *BoCCD4* possibly occurred in populations of the *B. oleracea* ancestors. Parallel phenotypic changes may be caused by different genetic changes, different changes at the same locus, and in some cases changes in the same nucleotide at the same locus [48–50], which may explain the phenomenon that some nonfunctional alleles of *BoCCD4* harbor combinations of different mutations, for example, the allele of the YL-1 inbred line harbors all three InDels, whereas the allele of the 02–12 inbred line harbors TE1 and the + 1094 deletion.

## Conclusions

In this study, the gene responsible for petal color in *B. oleracea* was mapped to a 207-kb fragment. A *carotenoid cleavage dioxygenase 4 (CCD4) gene*, *BoCCD4* was identified as a candidate. Sequence analysis revealed multiple mutations in the coding region of *BoCCD4* alleles of yellow-petal accessions. Overexpression of wild-type *BoCCD4* allele from 11-192 rescued the yellow-petal trait in YL-1, demonstrating that functional loss of *BoCCD4* resulted in the widespread yellow-petal *B. oleracea* accessions. This study provides insight into the formation of white/yellow petal color in *B. oleracea*.

## Methods

### Plant materials

*Brassica oleracea* lines YL-1 (yellow petal) and 11–192 (white petal) were described in a previous study [31]. These lines were used as parents to construct F_2_ and backcross (BC) populations for mapping *cpc-1* [31]. In this study, a larger F_2_ population comprising 1251 recessive (yellow petal) individuals was produced for map-based cloning of *cpc-1*.YL-1 was also used as acceptor plants for *Agrobacterium*-mediated transformation.

### Map-based cloning

Genomic DNA was extracted from fresh leaves of parents and F_2_ individuals using a modified CTAB (cetyl trimethylammonium bromide) protocol [31]. A set of insertion/deletion (InDel) markers (Additional file 3) around the previously reported mapping region was developed. Polymorphic markers between YL-1 and 11–192 were used to genotype all yellow-petal individuals of the F_2_ population. Polymerase chain reaction and polyacrylamide gel electrophoresis were performed following a previously described procedure [31]. Genetic and physical maps were constructed using MapDraw [51].

### Plasmid construction and functional complementation

For functional complementation, the coding sequence of *cpc-1* was amplified from white-petal parent 11–192 using the primer Bocpc-CDS. The fragment was subcloned into a modified binary vector pBWA(V) BS (reconstructed from pCAMBIA1301) driven by the CaMV35S promoter, and the hygromycin resistance gene was replaced with an herbicide resistance marker (Bar) to generate the construct Pro35S::BoCCD4. This construct was introduced into *Agrobacterium tumefaciens* strain GV3101 and transformed into yellow-petal parent YL-1 using the *Agrobacterium*-mediated transformation procedure for *B. oleracea* described by Yi et al. [52].

### Expression analysis of *BoCCD4*

Total RNA was extracted from plant tissues, including roots, stems, leaves, siliques, young buds, sepals, petals, pistils and anthers of YL-1 and 11–192 and petals of overexpressing lines using an RNAprep pure Plant Kit (TIANGEN, Beijing, China). Genomic DNA removing from the extracted RNA, first-strand cDNA synthesis and semi-quantitative reverse transcription-polymerase chain reaction (RT-PCR) were performed as previously described [53]. The primers for RT-PCR are listed in Additional file 3.

### Phylogenetic analysis

BLASTP searches were conducted using the amino acid sequence of BoCCD4 to search for homologs in the protein databases of the National Center for Biotechnology Information (NCBI) and Ensembl Plants (http://plants.ensembl.org). Protein sequence alignment was performed with MAFFT (v7.037) [54]. FastTree (LG + JTT model) was used to construct phylogenetic trees [55].

## Additional files

Additional file 1: Sequence alignment of the deduced BoCCD4 amino acid sequences from 11 to 192 and YL-1. The + 312-insertion in BoCCD4 of YL-1 alters the open reading frame and causes a premature stop codon, resulting in a predicted truncated 114-amino acid protein. (TIF 13823 kb)

Additional file 2: Polymorphisms of marker Bol035718D771 in parents and 13 recombinants. M, DNA ladder; P1, inbred line YL-1; P2, inbred line 11–192. (TIF 10174 kb)

Additional file 3: Primers used in this study for genetic mapping, gene amplification and RT-PCR. (DOCX 18 kb)

## Abbreviations

ABA: Abscisic acid
BC: Backcross
*CCD4*: *Carotenoid cleavage dioxygenase 4*
FLC: Flowering locus C
InDel: Insertion/deletion
NCBI: National Center for Biotechnology Information
NCED: 9-cis-epoxycarotenoid dioxygenase
SNPq: Single nucleotide polymorphism
UV: Ultraviolet
WGD: Whole-genome duplication
WGT: Whole-genome triplication
