# Supplementary figures and images for: Map-based cloning and characterization of BoCCD4, a gene responsible for white/yellow petal color in B. oleracea
Source: BMC Genomics. 2019 Mar 25;20:242. doi: 10.1186/s12864-019-5596-2 (PMC6434876; doi:10.1186/s12864-019-5596-2)

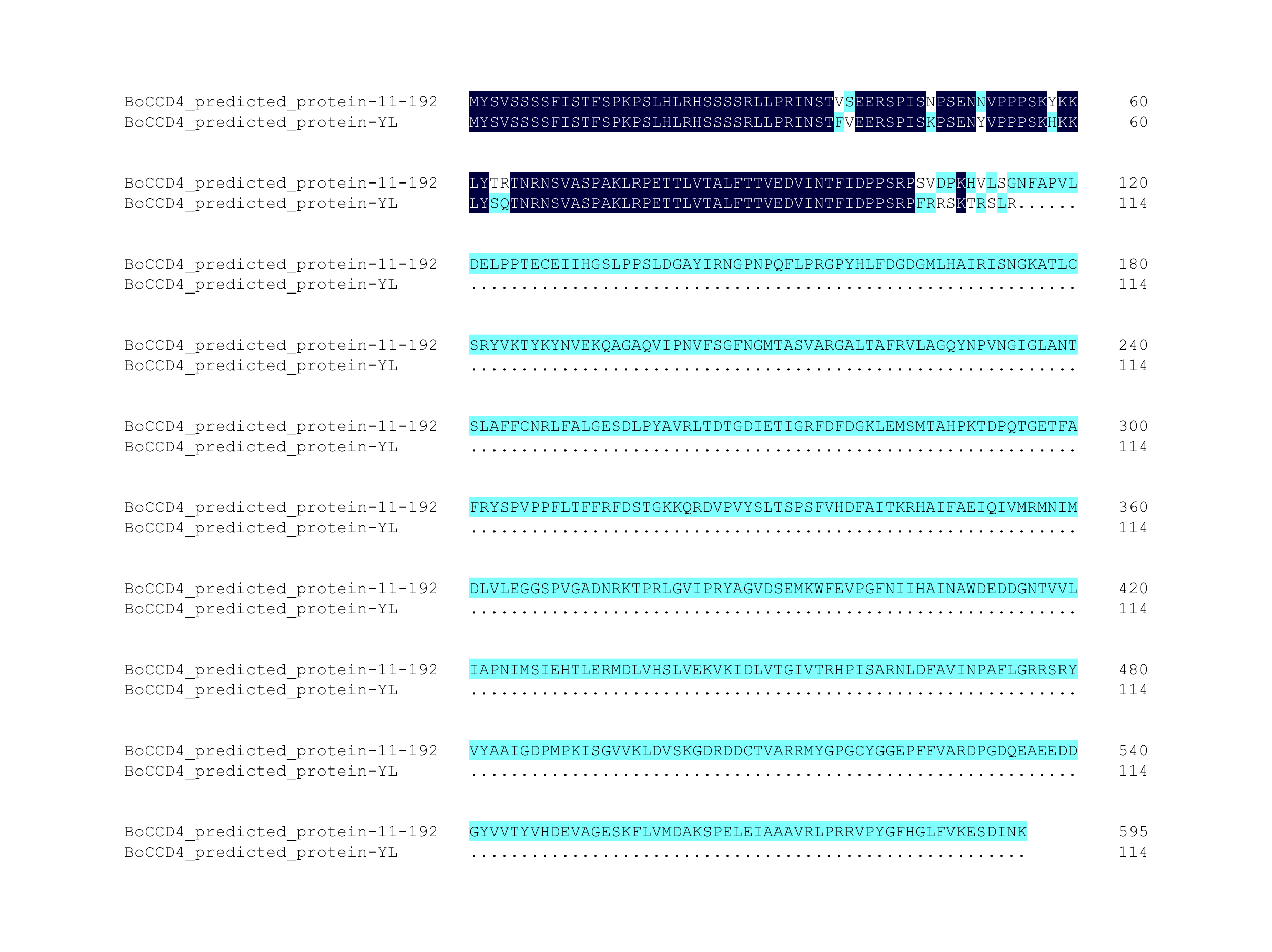

Supplement: Supplementary file 1 — Sequence alignment of the deduced BoCCD4 amino acid sequences from 11 to 192 and YL-1. The + 312-insertion in BoCCD4 of YL-1 alters the open reading frame and causes a premature stop codon, resulting in a predicted truncated 114-amino acid protein. (TIF 13823 kb) [file 12864_2019_5596_MOESM1_ESM.tif]

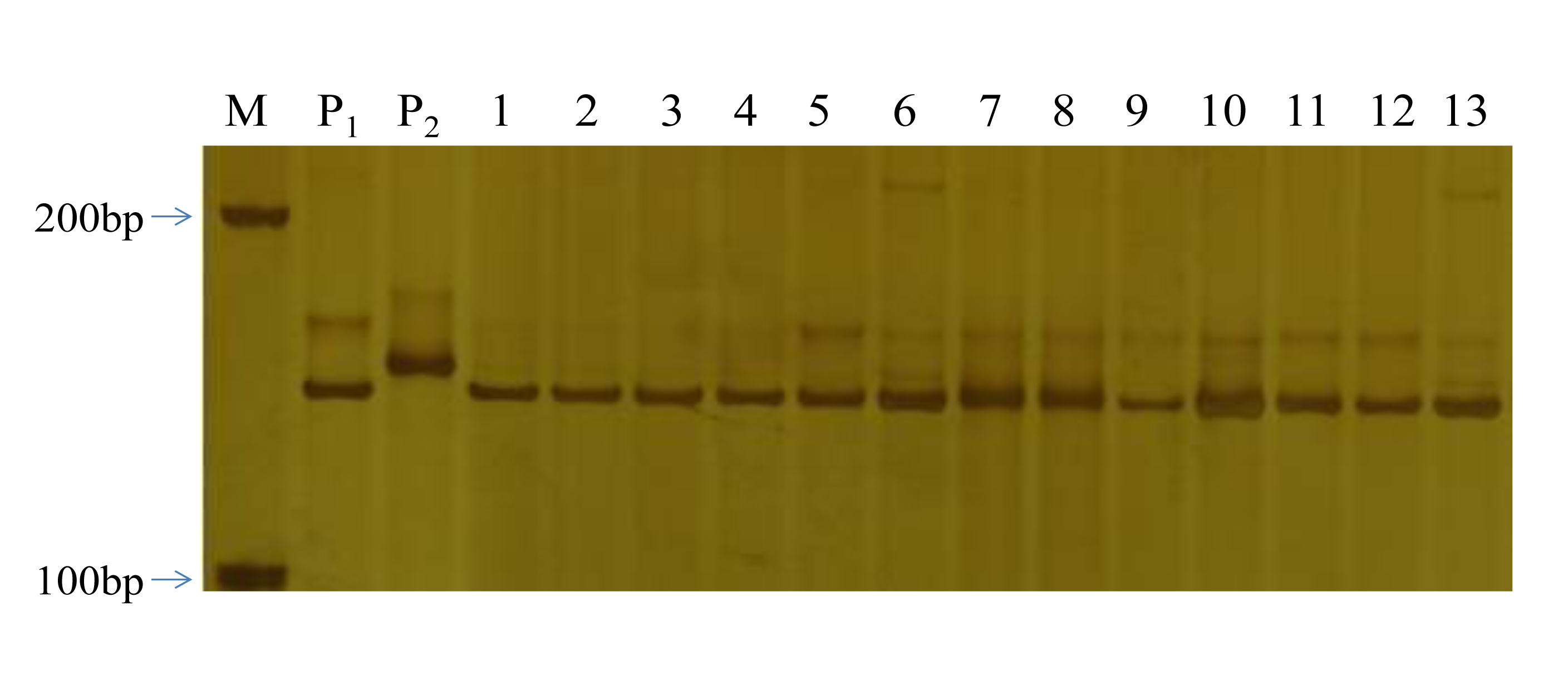

Supplement: Supplementary file 2 — Polymorphisms of marker Bol035718D771 in parents and 13 recombinants. M, DNA ladder; P1, inbred line YL-1; P2, inbred line 11–192. (TIF 10174 kb) [file 12864_2019_5596_MOESM2_ESM.tif]
