# Supplementary material for: Map-based cloning and characterization of BoCCD4, a gene responsible for white/yellow petal color in B. oleracea
Source: BMC Genomics. 2019 Mar 25;20:242. doi: 10.1186/s12864-019-5596-2 (PMC6434876; doi:10.1186/s12864-019-5596-2)
Supplement: Supplementary file 3 — Primers used in this study for genetic mapping, gene amplification and RT-PCR. (DOCX 18 kb) [file 12864_2019_5596_MOESM3_ESM.docx]

**Additional file 1** Primers used in this study for genetic mapping, gene amplification and RT-PCR.

| Primer name | Forward primer sequence(5'-3') | Reverse primer sequence(3'-5') |
| --- | --- | --- |
| M4008 | GTGAGCGATTCATATCCTGT | GAAGCGCATAGTTTGGTACT |
| M4015 | ATGAAACTGAGCCCATTAGA | CCTTTTGTTTTGTGTGTGTG |
| M4064 | AAGCACCAAACAGCTACACT | AAGTATCCCTTGATGGTTCA |
| M4122 | CACCTCAAGATTGGAAAAAG | AAAAACGAATCTGGTCAGTG |
| M4136 | TTATAATCAATGGGCCTGG | ATTTCAGCACCATTAAGGC |
| M4139 | GTTCCATGTTCTCTTTGGAA | AACTAGGGCTCGGTGTTTA |
| M4150 | TTCACATTGCCTGAATACTG | CCGTCTTCCACATTTTTG |
| M4161 | GCCTTAGAGCAACTCAATCA | AAATTGCTCTCCCACTAACC |
| M4212 | TCAGTCTCTTAGCAGCCC | CGATCCGAAGATATCTGAAC |
| M4219 | GAGGAATGCTTCAGATTTTG | TGTGGTTACATCTCGTTTGA |
| M4078 | CCATTTGCCAAAGTTGAAG | TGCTCTCCATTTGCTTCTT |
| M4080 | GCAATGACTCTATATCGCAA | AATGGCTACAAGCCTTTACA |
| M4085 | CATACACAAACACCCACTCA | GGTTTTGGATGGTAATGACA |
| M4089 | GTCTTCATTTGAATCCCAGA | TCAGGTGATCCATCTCTTTC |
| M4096 | GGTATTTCGCCTTTCAACTT | CCTGGTCCTATTCTCAATTTC |
| M4102 | TACATCATGGTCGTACCAAA | TCCCGAGTAGTCCCAACTAT |
| M4118 | TCATACGACAGCATCCTACA | TTTGACTAAACAAGGACCGT |
| M22 | AATCTCAGGGACTAACCAC | CAGTGACGCAATACTTGA |
| Boccd4RT TTCGCTTTCCGGTACAGTCC  TCATCTCGGAATCATCGCCGC1 | TTCGCTTTCCGGTACAGTCC  TTCGCTTTCCGGTACAGTCC  TTCGCTTTCCGGTACAGTCC | TCATCTCGGAATCATCGCCG |
| Bocpc-CDS | cagtGGTCTCacaacatgtactctgtttcttcctc | cagtGGTCTCatacattaaagcttattaatgtcgc |
| Bol035718D771 | GGCATTGGTTTGGCTAATAC | GCTCATCTCTAACTTCCCGT |
